# Blind Evaluation of Hybrid Protein Structure Analysis Methods based on Cross-Linking

**DOI:** 10.1016/j.tibs.2016.05.005

**Published:** 2016-07

**Authors:** Adam Belsom, Michael Schneider, Oliver Brock, Juri Rappsilber

**Affiliations:** 1 Wellcome Trust Centre for Cell Biology, University of Edinburgh, Edinburgh, EH9 3BF, UK; 2 Robotics and Biology Laboratory, Department of Electrical Engineering and Computer Science, Technische Universität Berlin, 10587 Berlin, Germany; 3 Chair of Bioanalytics, Institute of Biotechnology, Technische Universität Berlin, 13355 Berlin, Germany

## Abstract

Hybrid methods combine experimental data and computational modeling to analyze protein structures that are elusive to structure determination. To spur the development of hybrid methods, we propose to test them in the context of the CASP experiment and would like to invite experimental groups to participate in this initiative.

Determination of protein structure is an important prerequisite for understanding protein function, yet it remains one of the great scientific challenges of our time. One question: what are the tools that we would like to use? Light microscopes have been used for centuries to look at cellular structures, but we have not yet been able to develop a microscope powerful enough to observe or film a protein structure. However, we have been able to observe protein structure by interpreting physical measurements from X-ray diffraction, nuclear magnetic resonance (NMR) spectroscopy, and electron microscopy. These methods have provided us with most of the more than 110 000 structures in the Protein Data Bank (www.pdb.org) [1].

As we aim to chart the protein structural universe more widely and in more detail, established methods face some rough seas and potentially crippling challenges. Many proteins and complexes seem out of reach for existing methods, because they cannot be purified, are unstable, or their nature is intrinsically dynamic [2]. So-called ‘hybrid’ methods (methods that combine sparse and low-resolution experimental data and also high-resolution yet sparse structures, with computational structure modeling methods) could have the potential to overcome some of these limitations. The sparse, low-resolution data used in hybrid methods are by themselves insufficient to determine protein structure. However, their combination with computational structure modeling methods has been shown to enable the determination of complex model structures [3].

For hybrid methods to realize their potential, we must advance both the experimental methods and the corresponding computational methods. This development must occur in tandem so as to be able to achieve the most effective synergies between the strengths of both sides: the nature of the experimental data must determine what the most appropriate computational methods are, and the challenges of computational methods can guide the development of experimental methods.

One promising type of low-resolution experimental data exploitable by hybrid methods is obtained by cross-linking/mass spectrometry. Cross-linking/mass spectrometry is so promising because it appears to complement existing computational approaches very well [4]. Also, cross-linking/mass spectrometry is well established in the structural biology lexicon. It has been accepted and proven (by numerous successes) to elucidate the architecture of large protein complexes [5]. This has involved exogenous, homobifunctional cross-linkers that predominantly link lysine residues. Nevertheless, this robust and popular application has been limited in terms of the extent of detail that it reveals, which is largely a consequence of using selective cross-linkers. Solving entire structures appears to be out of reach. However, using a promiscuous and photoactivatable cross-linker instead may provide a fundamental change, at least for individual proteins. We validated the combination of high-density cross-linking data with controlled false discovery rates (FDR) and a conformational space search, because it enabled the determination of the structure of human serum albumin (HSA) domains with an RMSD to the X-ray structure of up to 2.5 Å, or 3.4 Å in the context of blood serum [4]. The generation and conjunction of high-density cross-linking/mass spectrometry data with computational structure modeling for *ab initio* structure prediction is very new and, consequently, needs to be questioned, tested, and developed further.

If we are going to spark the rapid development of both hybrid and component methods within hybrid methods, we need to target two important goals. First, we need to bring the experimental and computational communities together. This is important to allow cross-fertilization of ideas and to ensure that the latest developments in both fields are used. Second, we need to establish evaluation standards for hybrid methods to test their ability for structure determination on a highly rigorous but even playing field. Many hybrid approaches (and component methods) have been developed in the context of specific proteins and complexes and it is often not clear whether an approach will work for other proteins. To reach our two goals, we are proposing to now bring the two communities (experimental groups and protein-modeling experts) together in the context of the community-wide experiment, Critical Assessment of protein Structure Prediction (CASP) 6, 7, 8. We propose the use of CASP as a platform to facilitate progress in hybrid method development. To accomplish this goal, we are soliciting the participation of experimentalists to provide protein structure data for the upcoming CASP12, held in May–August 2016.

CASP has taken place every 2 years since 1994 and provides a stringent assessment platform of structure prediction methods. The organizers release protein sequences with known but unpublished structures to modeling groups, who can then test their ability to predict structures. The predicted structures are then evaluated by independent evaluation groups, with the goal of determining the most promising approaches and research directions. Importantly, this experiment is double blind to prediction groups, who do not know the protein structures, and evaluation groups, who do not know the origin of the predictions. Thus, CASP has established a rigorous assessment standard in the field of structure prediction that is unmatched in many areas of science and is considered to be one of the hallmark accomplishments of structural bioinformatics [9]. CASP was the platform for demonstrating the effectiveness of modern structure prediction methods, such as assembly from structural fragments, the detection of remote homologs, and, most recently, the use of evolutionary contacts 8, 10, 11. This rigorous testing of structure prediction methods spurred their development into a technology that is now routinely applied in protein engineering and drug design [12]. CASP also inspired similar efforts for docking of proteins into complexes (CAPRI [13]) and the automated testing of prediction servers (CAMEO [14]).

Following 20 years of purely computational work, in 2014 (CASP11) experimental data was made available to modeling groups to assist predictions for the first time [15]. Cross-linking/mass spectrometry succeeded in providing distance constraints for four proteins with a turnaround time of 2 weeks per protein. Here, CASP11 allowed us to test the readiness of the approach in a blind study and, at the same time, test the current value of cross-link data for structure prediction.

We identified between 201 and 381 unique residue pairs at an estimated 5% FDR, for the four proteins for which we provided data (Figure 1). This equates to between 0.63 and 1.20 cross-links per residue, which is comparable to that obtained in the HSA study (0.85 links per residue at 5% FDR). Initial results of CASP11 have suggested that improvements in *ab initio* structure prediction using cross-link data are slight [15]. Most significantly, however, CASP11 revealed some of the current limitations of cross-linking, defining areas in which the method must develop in the future. The observed cross-links were spread unevenly over the sequence. In addition, beta sheets had both a lack of links and weak definition of observed links over the structure. These cross-linking/mass spectrometry methodology limitations, identified during the course of CASP11, were not specific to this experiment; rather they are limitations that will be present for the whole field. By exposing these limitations, we hope that science is now better able to find the necessary solutions. Blind testing under the auspices of CASP, or a similar structure, allows method developers to clearly identify the most promising approaches as well as areas for future development and, perhaps more fundamentally, allows scientists at large to see the current maturity of the approaches as general methods.

## Call for Participation

We would like to open a call to all cross-linking/mass spectrometry groups who are interested in the further development of cross-linking/mass spectrometry technology and its ties to structure elucidation to consider participation in the next round of CASP. In addition, we would like to welcome all experimentalists who are able to produce low-resolution data for the development of hybrid structure determination. This could be heralded as a stepping-stone towards joining all experimental methods that provide some information on protein structures with the modeling community. Participation of experimental groups is a crucial element for successfully leveraging the full potential of this initiative. We should embrace this great opportunity to drive development of all aspects of protein structure modeling, whether it is the development of hybrid methods, modeling algorithms, or experimental data provision. We look forward to the development of novel tools in our toolbox, and the unprecedented discoveries in the protein universe that they will lead to.

For further details on how to participate in CASP12 as an experimentalist and to sign up, please contact: http://predictioncenter.org/casp12/registration.cgi.

## Figures and Tables

**Figure 1:**
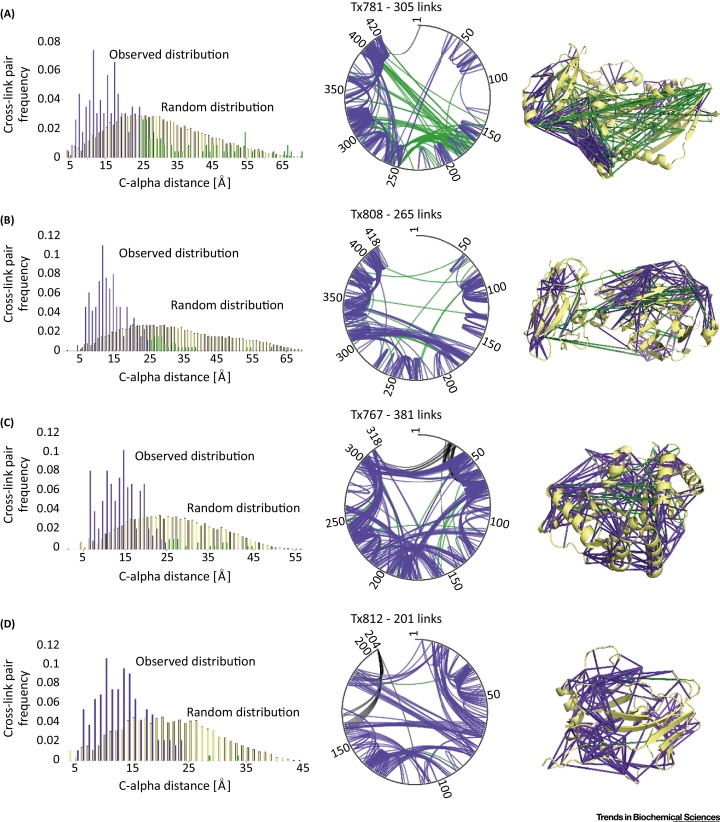
Cross-Linking/Mass Spectrometry Data Used in Critical Assessment of protein Structure Prediction 11 (CASP11). (A–D) Left panels show the C-alpha pair distance distribution of observed constraints at 5% false discovery rate (FDR) against the random constraint distribution. Middle panels show the cross-link networks for four CASP targets shown for estimated 5% FDR cut-off. Gray outer lines represent target sequences. Constraints missing from the crystal structure and, therefore, unverifiable are represented in black. Right panels show the observed constraints at 5% FDR against the X-ray structure. In all panels, constraints with Cα–Cα cross-linking distances less than 25 Å are shown in purple and constraints with distances 25 Å and over are shown in green. (A) Cross-linked residue pairs of Tx781 in Protein Data Bank (PDB)|4qan, *N* = 305. (B) Cross-linked residue pairs of Tx808 in PDB|4qhw, *N* = 265. (C) Cross-linked residue pairs of Tx767 in PDB|4qpv, *N* = 381. (D) Cross-linked residue pairs of Tx812 in crystal structure (structure not deposited in PDB), *N* = 201. Note, Tx781 was compromised during shipment and showed aggregates upon cross-linking that led to the pronounced presence of constraints not fitting the X-ray structure of that protein.
